# Pediatric firearm injury epidemiology at a level 1 trauma center from 2019 to 2021: including time of the COVID-19 pandemic

**DOI:** 10.1186/s40621-023-00448-3

**Published:** 2023-08-07

**Authors:** Cynthia Orantes, Hei Kit Chan, Daniel Walter, Summer Chavez, Irma T. Ugalde

**Affiliations:** 1 Department of Emergency Medicine, McGovern Medical School at The University of Texas Health Science Center at Houston (UTHealth), 6431 Fannin Street, JJL 475, Houston, Texas 77030 USA; 2 Texas Emergency Medicine Research Center, McGovern Medical School, Houston, USA; 3 Department of Health Systems and Population Health Sciences, Tilman J. Fertitta Family College of Medicine, University of Houston, 5055 Medical Circle Rm 1316, Houston, TX 77204 USA

**Keywords:** Pediatric trauma, COVID-19, Penetrating trauma, Epidemiology, Disparities

## Abstract

**Background:**

Firearms are a leading cause of death in children. The demand for firearms increased following COVID-19 “stay-at home orders” in March 2020, resulting in record-breaking firearm sales and background checks. We aim to describe the changes in pediatric firearm-related injuries, demographics, and associated risk factors at a Level 1 trauma center in Houston before and during the COVID 19 pandemic.

**Results:**

The total number of pediatric firearm-related injury cases increased during March 15th to December 31st, 2020 and 2021 compared to the same time period in 2019 (104 verses 89 verses 78). The demographic group most affected across years were males (87% in 2019 vs 82% in 2020 and 87% in 2021) between 14 and 17 years old (83% in 2019 vs 81% in 2020 and 76% in 2021). There was an increase in firearm injuries among black youth across all years (28% in 2019 vs 41% in 2020 vs 49% in 2021). Injuries in those with mental illness (10% in 2019 vs 24% in 2020 vs 17% in 2021), and injuries where the shooter was a known family member or friend (14% in 2019 vs 18% in 2020 vs. 15% in 2021), increased from 2019 to 2020.

**Conclusion:**

The total number of pediatric firearm-related injuries increased during the COVID-19 pandemic compared to the previous year despite a decline in overall pediatric emergency department visits. Increases in pediatric firearm-related injuries in already vulnerable populations should prompt further hospital initiatives including counseling on safe firearm storage, implementation of processes to identify children at risk for firearm injuries, and continued research to mitigate the risk of injury and death associated with firearms in our community.

## Background

Firearm-related injuries, the leading cause of death in children and adolescents in the USA, are an ongoing public health crisis (Fowler et al. 2022; Bleyer et al. 2022; Monuteaux et al. 2021). Between 2019 and 2020, firearm homicides increased 47% in children and adolescents less than 18 years old (Bell et al. (2021); Bernardin et al. (2021)). The demand for firearms sharply increased following “stay-at home orders” with the start of the COVID 19 pandemic (Fernández 2020; Hidalgo 2020), resulting in an estimated 2.6 million firearm sales in March 2020 alone (Brown et al. 2020) in the USA. These sales reached an all-time high in 2020, with 21 million firearms sold, close to a 49% increase since the year prior (Federal Bureau of Investigation (FBI) 2020; Safehome.org [Internet] 2023). Research has demonstrated that the leading driver for firearm acquisitions during the COVID-19 pandemic is for the protection of self and family (Khubchandani and Price 2021; Lyons et al. 2021). The most common concerns among firearm purchasers include increases in crime, feeling unsafe with masking mandates, struggles with obtaining supplies, and health and the economy (Khubchandani and Price 2021; Lyons et al. 2021). Firearm sales and background checks continued into 2021, with the second-highest year of firearms sales (Safehome.org [Internet] 2023). Additionally, in 2021 more firearms (approximately 1.6 million) were sold in Texas than any other state (Federal Bureau of Investigation (FBI) 2020; ABC-13 Houston [Internet] 2020). A surge in first-time firearm ownership has also been reported (Khubchandani and Price 2021; Lyons et al. 2021; ABC-13 Houston [Internet] 2020).

Prior studies observing pediatric firearm violence have focused on brief time periods during the pandemic within specific pediatric age ranges. These studies conclude that there was a rise in firearm injuries involving children particularly young children less than 12 years old in the USA during the first six to twelve months of the pandemic, which correlated with an increase in new firearm ownership (Gastineau et al. 2022; Cohen et al. 2022; Crichton et al. 2021). It is also well known that access to firearms in the home increases the risk for unintentional childhood injury, death, and suicide (Grossman et al. 2021; Cunningham et al. 2021; Schmidt et al. 2021). At the time of this study, there were no known published studies investigating pediatric firearm injuries in Houston, Texas. In Harris County, the county where this study was conducted, the second leading cause of death in children age 0–17 years old is attributed to firearms (Harris County Child Fatality Review Team [Internet] 2020). Harris County is the third largest county in the USA and the largest county in Texas. Additionally, “Permitless Carry” was signed into law in Texas in September 2021, eliminating the requirement for Texas residents to obtain a firearm license in order to carry a handgun and removing the requirement of proficiency testing. (Mulcahy and Sparber 2021). There is no longer verification that the firearm owner is trained or skilled in the handling of the firearm. Access to firearms has increased through increased sales and laws since the COVID-19 pandemic.

The objective of our study is to demonstrate the changes in prevalence and epidemiology of pediatric firearm-related injuries at a level 1 pediatric trauma center in Houston, Texas, during the COVID-19 pandemic compared to the year prior. We hypothesized that a nonnegligible increase in the number of pediatric firearm-related injuries occurred in 2020 and 2021 compared to the year prior at our trauma center.

## Results

There was a decline of 34% and 12% in total Pediatric Emergency Department visits in 2020 and 2021, respectively, compared to 2019. Table 1 depicts the changes in incidence of firearm injuries, patient demographics, and clinical characteristics during March 15th through December 31st of each study year. Notably, firearm injuries in black youth nearly doubled in 2020 and 2021, compared to 2019, nearly achieving statistical significance.

**Table 1:** Demographic and Clinical Characteristics of Firearm Injuries

	Total N = 271	2019 N = 78	2020 N = 104	2021 N = 89	*P *value ^a^
*Age, median (IQR *^*b*^***)***	16 (14–17)	16 (14–16)	16 (14–17)	15 (14–17)	0.45
*Age Categories, n (%)*					0.24
1–4	18 (6.6)	6 (7.7)	4 (3.8)	8 (9.0)	
5–9	13 (4.8)	3 (3.8)	3 (2.9)	7 (7.9)	
10–13	23 (8.5)	4 (5.1)	13 (12.5)	6 (6.7)	
14–17	217 (80.1)	65 (83.3)	84 (80.8)	68 (76.4)	
*Sex, n (%)*					0.52
Male	230 (84.9)	68 (87.2)	85 (81.7)	77 (86.5)	
Female	41 (15.1)	10 (12.8)	19 (18.3)	12 (13.5)	
*Race, n (%)*					0.08
White	38 (14.0)	15 (19.2)	15 (14.4)	8 (9.0)	
Black	109 (40.2)	22 (28.2)	43 (41.3)	44 (49.4)	
Asian	3 (1.1)	1 (1.3)	2 (1.9)	0 (0.0)	
Other	114 (42.1)	37 (47.4)	40 (38.5)	37 (41.6)	
*Ethnicity, n (%)*					0.90
Hispanic	77 (28.4)	23 (29.5)	30 (28.8)	24 (27.0)	
Non-Hispanic	170 (62.7)	46 (59.0)	68 (65.4)	56 (62.9)	
Hospital charges ($1,000), median (IQR ^b^***)***	$68 k ($24 k–$149 k)	$63 k ($33 k–$122 k)	$86 k ($20 k–$171 k)	$57 k ($22 k–$107 k)	0.35
Hospital length of stay (day), median (IQR ^b^***)***	3 (1–7)	3 (2–6)	3 (1–7)	2 (1–7)	0.28
ED Length of stay (hour), median (IQR ^b^***)***	4.0 (1.6–6.7)	3.9 (1.4–6.4)	3.4 (1.0–6.2)	4.3 (2.8–7.0)	0.06
*GCS, n (%)*					0.15
> 13	218 (80.4)	60 (76.9)	80 (76.9)	78 (87.6)	
≤ 13	51 (18.8)	17 (21.8)	23 (22.1)	11 (12.4)	
*ISS, n (%)*					0.70
< 16	192 (70.8)	54 (69.2)	72 (69.2)	66 (74.2)	
≥ 16	79 (29.2)	24 (30.8)	32 (30.8)	23 (25.8)	
*ED disposition, n (%)*					0.79
Floor/OBS	102 (37.6)	28 (35.9)	36 (34.6)	38 (42.7)	
ICU/IMU	45 (16.6)	13 (16.7)	19 (18.3)	13 (14.6)	
OR	87 (32.1)	27 (34.6)	33 (31.7)	27 (30.3)	
Deceased	15 (5.5)	5 (6.4)	8 (7.7)	2 (2.2)	
Discharged to home	21 (7.7)	5 (6.4)	8 (7.7)	8 (9.0)	
Against medical advice	1 (0.4)	0 (0.0)	0 (0.0)	1 (1.1)	

^a^*P* values of age, hospital charges, hospital length of stay, and ED length of stay were derived from Kruskal–Wallis test. *P* values of sex, ethnicity, GCS, and ISS were derived from Pearson’s Chi-square test. P values of age category, race, and ED disposition were derived from Fisher’s exact test

^b^IQR stands for interquartile range

Bold values indicate a percentage change with a 95% confidence interval that does not cross 0

Characteristics related to individual patient injuries are shown in Table 2. Back/flank injuries (190.1%, 95% CI 30.4–545.5%) and head injuries (269.2%, 95% CI 27.4–970.4%) increased significantly from 2019 to 2020. The greatest proportion of incidents had homicidal/violent intent and occurred away from home; however, this did not significantly change over time. Incident demographics related to the firearm incident are shown in Table 3. From 2019 to 2020, there was a notable decrease (− 79.5%, 95% CI − 95.4– − 7.9%) in adult supervision.

**Table 2:** Firearm injury patterns and severity

	Total *N* = 271	2019 *N* = 78	2020 *N* = 104	2021 *N* = 89	Percentage change from 2019 to 2020 ^a^ (95% confidence interval)	Percentage change from 2019 to 2021 ^a^ (95% confidence interval)	Estimated annual percentage change ^a^ (95% confidence interval)
*Injury location, n (%)*							
Head/Neck	72 (26.6)	22 (28.2)	30 (28.8)	20 (22.5)	25.9% (− 22.5–104.4%)	− 12.7% (− 48.7–48.5%)	− 6.1% (− 25.9–19.1%)
Chest	36 (13.3)	15 (19.2)	11 (10.6)	10 (11.2)	− 32.3% (− 67.5–41.1%)	− 36.0% (− 70.0–36.4%)	− 20.9% (− 46.9–17.7%)
Back/Flank	38 (14.0)	7 (9.0)	22 (21.2)	9 (10.1)	**190.1% (30.4–545.5%)**	23.4% (− 51.6–214.5%)	6.2% (− 21.7–44.0%)
Abdomen	40 (14.8)	11 (14.1)	13 (12.5)	16 (18.0)	9.1% (− 49.4–135.2%)	39.6% (− 32.0–186.6%)	18.8% (− 17.8–71.7%)
Pelvis/Groin	24 (8.9)	7 (9.0)	7 (6.7)	10 (11.2)	− 7.7% (− 66.0–150.8%)	37.1% (− 47.1–255.9%)	18.8% (− 29.0–98.7%)
Spine	15 (5.5)	5 (6.4)	8 (7.7)	2 (2.2)	47.7% (− 49.5–331.8%)	− 61.6% (− 92.3–92.3%)	− 28.2% (− 57.3–20.7%)
Extremity	147 (54.2)	44 (56.4)	59 (56.7)	44 (49.4)	23.8% (− 6.5–63.8%)	− 4.0% (− 27.9–27.9%)	− 2.0% (− 14.1–11.8%)
*Injury type, n (%)*							
Superficial Injury	2 (0.7)	0 (0.0)	0 (0.0)	2 (2.2)	–	–	–
Open Wounds	58 (21.4)	14 (17.9)	21 (20.2)	23 (25.8)	38.5% (− 27.2–163.3%)	57.7% (− 16.7–198.5%)	24.5% (− 8.0–68.6%)
Unspecified	4 (1.5)	0 (0.0)	3 (2.9)	1 (1.1)	–	–	45.0% (− 28.3–193.1%)
Fractures	104 (38.4)	32 (41.0)	36 (34.6)	36 (40.4)	3.8% (− 30.7–55.6%)	8.0% (− 27.2–60.2%)	3.9% (− 14.7–26.6%)
Amputations	1 (0.4)	1 (1.3)	0 (0.0)	0 (0.0)	–	–	–
Internal Organs	64 (23.6)	24 (30.8)	20 (19.2)	20 (22.5)	− 23.1% (− 55.6–33.3%)	− 20.0% (− 52.9–36.0%)	− 11.0% (− 32.6–17.6%)
Blood Vessels	8 (3.0)	2 (2.6)	5 (4.8)	1 (1.1)	130.8% (− 54.0–1056.6%)	− 52.0% (− 95.6–420.7%)	− 19.2% (− 57.6–54.1%)
Nerves	4 (1.5)	1 (1.3)	3 (2.9)	0 (0.0)	177.0% (− 70.7%–2517.4%)	–	− 33.8% (− 67.2–33.8%)
Head Injury	26 (9.6)	4 (5.1)	16 (15.4)	6 (6.7)	**269.2% (27.4–970.4%)**	44.0% (− 57.7–390.6%)	10.3% (− 23.0–57.9%)
*Disability, n (%)*							
Yes	48 (17.7)	13 (16.7)	19 (18.3)	16 (18.0)	34.9% (− 28.1–153.3%)	18.2% (− 38.8–128.1%)	7.8% (− 20.4–46.0%)
No	222 (81.9)	65 (83.3)	85 (81.7)	72 (80.9)	**20.7% (1.1–44.1%)**	6.3% (− 10.2–25.9%)	2.8% (− 5.1–11.5%)
*Death, n (%)*							
Yes	28 (10.3)	10 (12.8)	12 (11.5)	6 (6.7)	10.8% (− 50.8–149.3%)	− 42.4% (− 78.0–50.6%)	− 21.4% (− 47.8–18.3%)
No	243 (89.7)	68 (87.2)	92 (88.5)	83 (93.3)	**24.9% (5.2–48.3%)**	**17.2% (0.9–36.0%)**	**7.7% (0.3–15.7%)**

^a^Percentage changes were estimated with Poisson regression model with year as the main predictor

CI stands for confidence interval

Bold values indicate a percentage change with a 95% confidence interval that does not cross 0

**Table 3:** Firearm Injury Incident Characteristics

	Total *N* = 271	2019 *N* = 78	2020 *N* = 104	2021 *N* = 89	Percentage change from 2019 to 2020 ^a^ (95% confidence interval)	Percentage change from 2019 to 2021 ^a^ (95% confidence interval)	Estimated annual percentage change ^a^ (95% confidence interval)
*Intent, n (%)*							
Accidental	58 (21.4)	21 (26.9)	23 (22.1)	14 (15.7)	1.1% (− 39.6–69.3%)	− 36.0% (− 66.3–21.5%)	− 18.6% (− 39.1–8.7%)
Self-harm	17 (6.3)	5 (6.4)	6 (5.8)	6 (6.7)	10.8% (− 67.8–281.0%)	15.2% (− 63.3%–261.8%)	7.2% (− 39.1%–88.7%)
Homicide/Violence	170 (62.7)	46 (59.0)	62 (59.6)	62 (69.7)	24.4% (− 5.6%–63.9%)	**29.4% (0.1–67.3%)**	**13.2% (0.1–28.1%)**
Police Violence	1 (0.4)	1 (1.3)	0 (0.0)	0 (0.0)	–	–	–
Not Documented	26 (9.6)	6 (7.7)	12 (11.5)	8 (9.0)	84.6% (27.0–367.1%)	28.0% (− 53.4–251.5%)	10.3% (− 27.1–66.6%)
*Location, n (%)*							
At Home	69 (25.5)	19 (24.4)	29 (27.9)	21 (23.6)	40.9% (− 15.4–134.7%)	6.1% (− 38.6–83.4%)	2.4% (− 19.7–30.7%)
Not At Home	176 (64.9)	54 (69.2)	64 (61.5)	58 (65.2)	9.4% (− 15.1–41.0%)	3.1% (− 18.1–29.8%)	1.4% (− 9.3–13.5%)
*Relationship of shooter to patient, n (%)*							
Self	42 (15.5)	14 (17.9)	14 (13.5)	14 (15.7)	− 7.7% (− 54.1–85.6%)	− 4.0% (− 50.8–87.2%)	− 2.0% (− 30.3–37.8%)
Family Member	20 (7.4)	7 (9.0)	7 (6.7)	6 (6.7)	− 7.7% (− 66.0–150.8%)	− 17.7% (− 71.0–133.7%)	− 9.2% (− 45.9–52.3%)
Friend	25 (9.2)	5 (6.4)	12 (11.5)	8 (9.0)	121.5% (− 18.1–499.3%)	53.6% (-47.4–348.7%)	17.9% (-22.3–78.7%)
Police	1 (0.4)	1 (1.3)	0 (0.0)	0 (0.0)	–	–	–
Stranger	146 (53.9)	40 (51.3)	56 (53.8)	50 (56.2)	29.2% (− 6.0–77.7%)	20.0% (− 10.5–60.9%)	8.8% (− 5.1–24.8%)
Unknown	37 (13.7)	11 (14.1)	15 (14.4)	11 (12.4)	25.9% (− 38.2–156.2%)	− 4.0% (− 55.7–107.8%)	− 2.0% (− 31.1–39.3%)
*Adult supervision, n (%)*							
Yes	18 (6.6)	9 (11.5)	2 (1.9)	7 (7.9)	− **79.5% (**− **95.4–7.9%)**	− 25.3% (− 70.7–90.3%)	− 17.4% (− 56.4–56.4%)
No	67 (24.7)	20 (25.6)	30 (28.8)	17 (19.1)	38.5% (− 14.8–125.0%)	− 18.4% (− 54.5–46.4%)	− 8.5% (− 28.5–17.1%)
Not Applicable	167 (61.6)	46 (59.0)	61 (58.7)	60 (67.4)	22.4% (− 6.7–60.6%)	25.2% (− 2.5–60.8%)	11.4% (− 1.2–25.6%)
*Firearm storage documentation, n (%)*							
Yes	15 (5.5)	2 (2.6)	3 (2.9)	10 (11.2)	38.5% (− 76.3–708.2%)	**380.0% (8.5–2022.7%)**	**146.7% (12.2–442.7%)**
No	50 (18.5)	18 (23.1)	19 (18.3)	13 (14.6)	− 2.6% (− 45.4%–73.9%)	− 30.7% (− 64.6%–35.8%)	− 16.0% (− 38.6–14.9%)
Not Applicable	136 (50.2)	46 (59.0)	60 (57.7)	30 (33.7)	20.4% (− 8.9%–59.1%)	− **37.4% (**− **56.2–10.6%)**	− **18.2% (**− **29.6–5.1%)**
*Firearm storage lock, n (%)*	*N* = 35	*N* = 9	*N* = 16	*N* = 10			
Yes	2 (5.7)	1 (11.1)	0 (0.0)	1 (10.0)	–	–	–
No	33 (94.3)	8 (88.9)	16 (100.0)	9 (90.0)	84.6% (− 16.0–305.8%)	8.0% (− 56.0–165.0%)	2.6% (− 28.0–46.4%)
*Firearm storage ammunition separation, n (%)*	*N* = 35	*N* = 9	*N* = 16	*N* = 10			
Yes	1 (2.9)	1 (11.1)	0 (0.0)	0 (0.0)	–	–	–
No	34 (97.1)	8 (88.9)	16 (100.0)	10 (100.0)	84.6% (− 16.0–305.8%)	20.0% (− 49.9–187.5%)	7.2% (− 24.7–52.6%)
*Law enforcement involvement, n (%)*							
Yes	211 (77.9)	68 (87.2)	77 (74.0)	66 (74.2)	4.5% (− 12.6–25.0%)	− 6.8% (− 23.7–13.7%)	− 3.4% (− 12.3–6.4%)
No	38 (14.0)	5 (6.4)	10 (9.6)	23 (25.8)	84.6% (− 33.9–415.5%)	**341.6% (75.7–1009.7%)**	**117.3% (40.7–235.6%)**
*Law enforcement action, n (%)*							
Yes	27 (10.0)	6 (7.7)	9 (8.7)	12 (13.5)	38.5% (− 48.3–270.5%)	92.0% (− 24.0–385.2%)	38.6% (− 11.8–117.8%)
No	43 (15.9)	4 (5.1)	2 (1.9)	37 (41.6)	− 53.8% (− 91.3–145.3%)	**788.0% (231.7–2277.1%)**	**391.6% (139.0–911.1%)**
*Child protection services involvement, n (%)*							
Yes	72 (26.6)	21 (26.9)	24 (23.1)	27 (30.3)	5.5% (− 37.2–77.1%)	23.4% (− 23.8–99.8%)	11.3% (− 12.8–42.2%)
No	189 (69.7)	54 (69.2)	73 (70.2)	62 (69.7)	24.8% (− 0.7–56.8%)	10.2% (− 13.2–40.0%)	4.5% (− 6.6–17.0%)
*Child protection services action, n (%)*							
Yes	14 (5.2)	6 (7.7)	4 (3.8)	4 (4.5)	− 38.5% (− 85.0–152.0%)	− 36.0% (− 81.2–118.1%)	− 21.4% (− 59.5–52.6%)
No	45 (16.6)	14 (17.9)	11 (10.6)	20 (22.5)	− 27.5% (− 64.8–49.5%)	37.1% (− 25.0–150.6%)	20.3% (− 14.9–70.1%)

^a^Percentage changes were estimated with Poisson regression model with year as the main predictor

CI stands for confidence interval

Bold values indicate a percentage change with a 95% confidence interval that does not cross 0

**Fig. 1:**
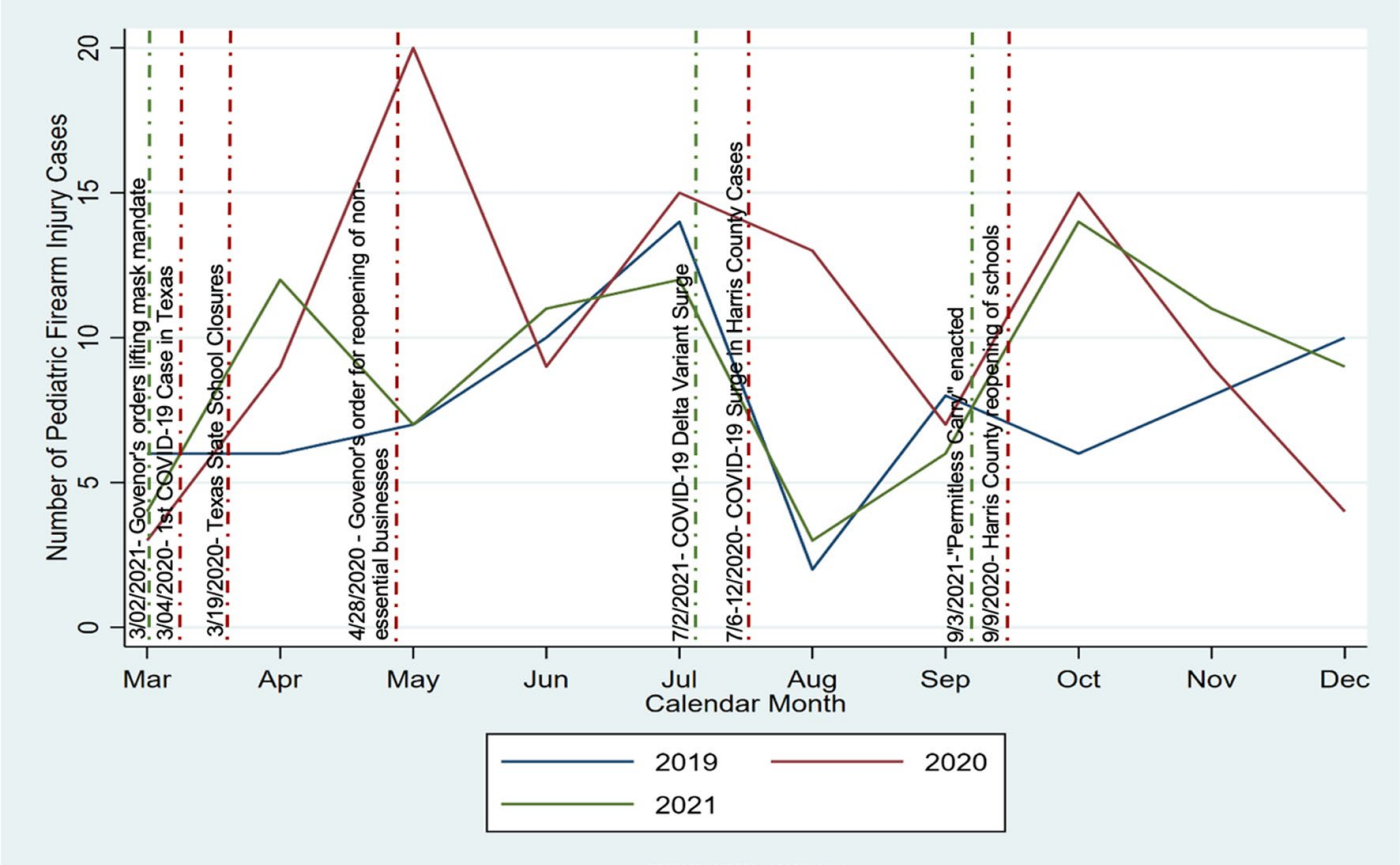
The number of pediatric firearm injuries are plotted on the y axis from March through December, 2019–2021 along the x axis, with each year represented by a different color line (2019, blue; 2020, red; 2021, green). Texas state mandates for school closures, re-openings of nonessential businesses and schools, lifting of mask mandates, the enactment of “Permitless Carry” as well as COVID surges and hospitalizations are shown in vertical lines by date of occurence

In Fig. 1, counts of pediatric firearm injury cases by month are plotted across years. Firearm injuries rose or peaked in 2020 and 2021 following city business re-openings, school openings, the lifting of mask mandates, and the enactment of “Permitless Carry.” Firearm injuries decreased initially following surges in COVID 19 cases and record-breaking hospitalizations in the county across both pandemic years before they rose again.

**Fig. 2:**
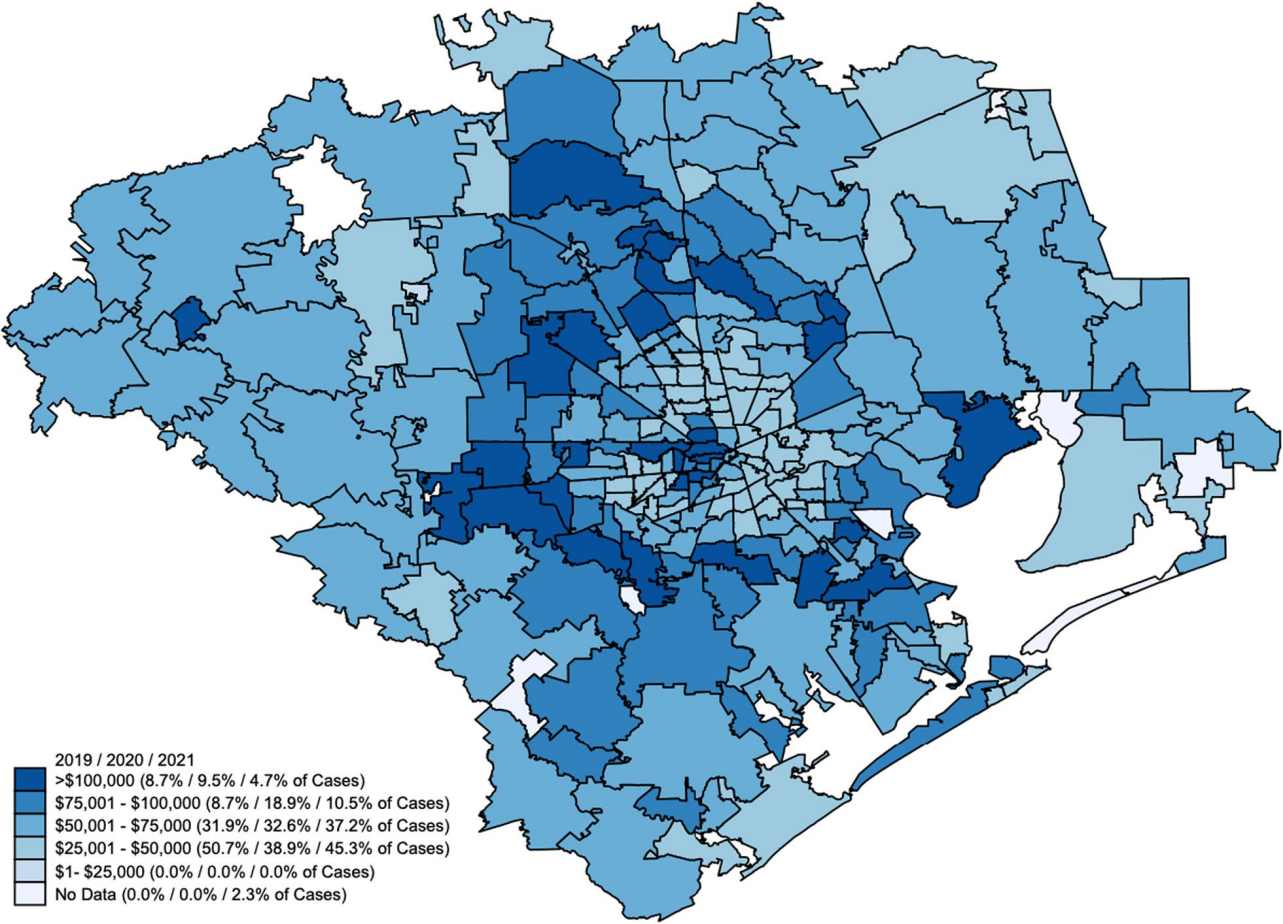
Mapping of the distribution of the household income brackets for children with firearm injuries based on zip code demographics with the darkest hue representing the highest income bracket and each subsequent lighter hue representing lower income brackets.  Percentages of firearm injuries for each income bracket in 2019, 2020, and 2021 are shown in parentheses

Figure 2 demonstrates the distribution of pediatric firearm injuries in Houston and the surrounding areas by income bracket. While most injuries occurred in children from households with an income bracket of $25,000 to $50,000, there was an increase in cases in the $75,000–100,000 income bracket in 2020, with percentage of injuries doubling that year compared to 2019. The rise was much less pronounced in 2021 compared to 2019.

Patient socioeconomic risk factors for firearm violence are reported in Table 4. While most risk factors did not change significantly, cases with history of mental illness in patients showed an increase of 188.5% (95% CI 36.6–509.0%) in 2020 compared to 2019 with a less drastic rise of 80.0% (95% CI − 20.5–307.8%) in 2021 compared to 2019. Incidents occurring in single parent households increased by 256.0% (95% CI 110.1–503.5%) in 2020 compared to 2019 and an increase of 297.7% (95% CI 139.9–559.3%) in 2021 compared to 2019, resulting in an estimated annual percentage increase of 71.7% (95% CI: 44.2–104.5%).

**Table 4:** Patient-Related Risk Factors Associated with Firearm Injuries

	Total *N* = 271	2019 *N* = 78	2020 *N* = 104	2021 *N* = 89	Percentage change from 2019 to 2020 ^a^ (95% confidence interval)	Percentage change from 2019 to 2021 ^a^ (95% confidence interval)	Estimated annual percentage change ^a^ (95% confidence interval)
*History, n (%)*							
Prior related firearm injury	12 (4.4)	2 (2.6)	7 (6.7)	3 (3.4)	223.1% (− 30.9–1409.9%)	44.0% (–75.3–740.1%)	11.3% (–36.0–93.7%)
Drug, gang, or violence involvement	38 (14.0)	15 (19.2)	15 (14.4)	8 (9.0)	–7.7% (–52.7–80.0%)	–48.8% (–76.9–13.6%)	–26.4% (–48.3–4.8%)
Substance use in patient or parent	129 (47.6)	36 (46.2)	48 (46.2)	45 (50.6)	23.1% (–13.8–75.7%)	20.0% (–14.0–67.5%)	9.0% (–7.0–27.9%)
Mental illness in patient	48 (17.7)	8 (10.3)	25 (24.0)	15 (16.9)	**188.5% (36.6–509.0%)**	80.0% (–20.5–307.8%)	22.7% (–8.3–64.1%)
Mental illness in parent	17 (6.3)	8 (10.3)	5 (4.8)	4 (4.5)	–42.3% (–80.3–68.7%)	–52.0% (–84.9–52.9%)	–32.2% (–62.9–23.8%)
Domestic violence/IPV/Abuse in patient or parent	22 (8.1)	5 (6.4)	5 (4.8)	12 (13.5)	–7.7% (–72.2–206.5%)	130.4% (–14.7–522.6%)	62.6% (–7.2–185.2%)
Chronic illness	24 (8.9)	8 (10.3)	8 (7.7)	8 (9.0)	–7.7% (–65.7–148.7%)	–4.0% (–62.0–142.6%)	–2.0% (–39.0–57.3%)
Food Insecurity	25 (9.2)	4 (5.1)	12 (11.5)	9 (10.1)	176.9% (–8.9–742.0%)	116.0% (–30.6–572.0%)	33.7% (-12.1–103.4%)
*Single parent household, n (%)*							
Yes	126 (46.5)	14 (17.9)	54 (51.9)	58 (65.2)	**256.0% (110.1–503.5%)**	**297.7% (139.9–559.3%)**	**71.7% (44.2–104.5%)**
No	54 (19.9)	10 (12.8)	24 (23.1)	20 (22.5)	**121.5% (13.8–331.2%)**	92.0% (–6.1–292.8%)	30.6% (–2.4–74.7%)

^a^Percentage changes were estimated with Poisson regression model with year as the main predictor

CI stands for confidence interval

Bold values indicate a percentage change with a 95% confidence interval that does not cross 0

## Discussion

Pediatric firearm injuries increased in 2020 and 2021 compared to 2019 at our trauma center in Houston. The incidence of firearm injuries in Black children doubled in 2020 and 2021 from 2019. Interestingly, the firearm injury peaks in our community were associated with local city re-openings mirroring prior studies, and the lifting of mask mandates (Ernst et al. 2020; Svitek 2020; Office of the Texas Governor | Greg Abbott [Internet] 2021), but decreased immediately following COVID surges and hospitalizations before rising again. Firearm injuries also rose following the passage of House Bill 127, which would be known as “Permitless Carry” (Svitek 2021). Our study investigated the immediate time period after the COVID pandemic in addition to a year out. Our findings parallel previous reports demonstrating increases in pediatric firearm-related injuries following the reopening of schools and businesses after the enaction of the COVID-19 stay-at-home orders (Abdallah et al. 2022; Bell et al. 2022; Hatchimonji et al. 2021).

While increased pediatric firearm injuries are linked to increased firearm sales and access (Donnelly et al. 2021a, b), other factors related to the COVID-19 pandemic may have contributed. The COVID-19 pandemic resulted in unprecedented social and economic disruptions such as the loss of jobs, the threat of eviction, record numbers of those living in extreme poverty, closures of schools, and isolation (Human Rights Watch [Internet] 2021). Pediatric firearm violence may have been affected by the higher prevalence of those living with depression and anxiety triggered by the COVID-19 pandemic (World Health Organization [Internet] 2022; Slomski 2022). Isolation, scarcity of resources, and fear of death for self and others may have contributed. While our data collection of potential risk factors such as a history of mental illness, food insecurity, and substance abuse was limited due to lack of consistent documentation, youth firearm exposure has been linked to higher rates of post-traumatic stress, mood disorders such as depression and anxiety, as well as substance abuse (Ranney et al. 2021). Studies have also shown that racial and ethnic minorities have been disproportionately affected by the COVID-19 pandemic, with millions of Black and Hispanic American’s falling into poverty more than twice the rate when compared to White Americans (Human Rights Watch [Internet] 2021).

Others have demonstrated the link between pediatric firearm injuries to new firearm acquisitions during the COVID-19 pandemic (Cohen et al. 2022). They report that firearm injuries in young children are often unintentional, compared to adolescents which are more often intentional injuries (Bernardin et al. 2022; Nordin et al. 2021). Our study redemonstrated similar intent categorizations by age, but shows a clear exacerbation during the pandemic. Over a quarter of all firearm injuries across years were accidental or self-harm intent. The American Academy of Pediatrics recognizes firearm violence prevention advocacy as one of their priorities, and has most recently stated that physician and staff training programs on firearm safety conversations can be lifesaving (American Academy of Pediatrics [Internet] 2021). Initiatives focused on increasing the general population’s awareness regarding proper storage of firearms and ammunition, the usage of firearm locks, as well as increased training of emergency departments, first responders and medical homes on firearm injury prevention are necessary (American Academy of Pediatrics [Internet] 2021; Brewer et al. 2021).

Our review demonstrated that there may have been possible missed opportunities for safety screening and storage counseling, due to the limited or missing documentation in the electronic medical record (EMR). This is an area of quality improvement to work together with other stakeholders in the hospital to improve processes related to patient intake and anticipatory guidance. Our study suggests that further investigation on a statewide database of pediatric firearm-related injuries following recent legislative changes may also be beneficial for planning local, county, or state interventions aimed at preventing pediatric firearm injuries.

### Limitations

Our findings are from a single center, retrospective cross-sectional study, and thus limit generalizability and external validity. However, they mirror prior reports across the nation. Furthermore, another limitation was having one year as the historical control to compare with the pandemic study period. Yet, through prior work, we know the average number of pediatric firearm injuries was nearly a third lower in 2017–2019 when compared to the average number in 2020–2021, resulting in a similar conclusion. Our review of the risk factors within our study was largely limited by the lack of documentation in the EMR, which led to smaller sample sizes and wider confidence intervals in Tables 3 and 4. Other potential inconsistencies in demographic data may have occurred during data entry or during patient intake. Lastly, while our sample size was robust, our study was limited to one of the two level-1 pediatric trauma centers in the city of Houston, Texas, which may have missed some of the firearm injuries during the study periods. However, our center has historically seen the majority of these penetrating injuries.

## Conclusion

The COVID-19 pandemic has forced families to face unprecedented challenges that continue to persist. Our institution experienced a rise in pediatric firearm-related injuries despite a decrease in total pediatric emergency department visits. This paralleled the increase in background checks and firearm sales across our city and nation following COVID-19 stay-at-home orders (Federal Bureau of Investigation (FBI) 2020; Lyons et al. 2021; ABC-13 Houston [Internet] 2020). Our study demonstrated sudden increases in firearm violence over time in relation to the COVID-19 pandemic. Attention should therefore be placed to further understand how psychosocial stressors, family and community dynamics, and mental health may contribute to pediatric firearm-related injury. These represent opportunities for future study. Healthcare workers should remain vigilant of potential modifiable risk factors associated with injury, such as domestic violence, mental illness, access to firearms, and storage practices. We believe the increases in pediatric firearm-related injuries should prompt national and statewide initiatives to help mitigate the risk of firearm-related injury and death especially as access to firearms may continue to increase following recent legislative changes.

## Methods

### Study design, setting, and population

We conducted a retrospective cross-sectional review of pediatric firearm-related visits at the study site from March 15th to December 31st of 2019–2021, examining demographics and visit characteristics in addition to associated risk factors. The study was conducted at a busy urban and quaternary level 1 trauma center in the southern USA. The catchment area is approximately 2.3 million children, where nearly 10% of the population are under the age of five years (U.S. Census Bureau 2022).

We adhered to the methods set by Worster et al. regarding the standards of emergency medical review (Worster et al. 2021). The inclusion and exclusion criteria for case selection were defined prior to data abstraction. We included patients from our institutional trauma registry less than 18 years presenting for initial evaluation or who were transferred to the emergency department with a firearm-related injury. Approval for this study was secured from the University of Texas Health (UTH) Sciences Committee for Protection of Human Subjects Institutional Review Board (IRB). The registry follows National Trauma Data Standards (NTDS) data dictionary and uses a third-party software called Traumabase (ESO, Austin, Texas). The trauma registry has internal data verification processes to ensure accuracy. We obtained median income data from Harris County and the surrounding area from the US Census Bureau (US Census Bureau [Internet] 2022). Firearm injuries were mapped using census population and income data.

### Measurements and outcomes

In this study, we investigated changes in the epidemiology of pediatric-related firearm injuries presenting to the pediatric ED from March 15th, 2020 to December 31st, 2020, and from March 15th, 2021 to December 31st, 2021, referencing the same time period in 2019 as the historical control. The dependent variable was the incidence of pediatric-related firearm injuries during the two years of the pandemic compared to the prior year as well as the frequencies in ED disposition categories, disability and death, intent of shooter, location of incident, relationship of shooter to patient, firearm and ammunition storage, presence of adult supervision, and risk factors. Risk factors included history of prior gun injury, drug and or gang violence, substance use, mental illness in patient or family member, history of domestic or intimate partner violence, history of chronic illness and belonging to single parent household. For all participants, we collected demographic information including age, race, ethnicity, sex, and patient zip codes. Visit characteristics including ED disposition, Glasgow Coma Score (GCS), ED length of stay (LOS), and hospital LOS. Documentation from social workers, pediatric forensic medical providers, and psychiatrists were reviewed in the EMR. Psychosocial and behavioral history were limited to self-reporting by the patient and or their guardian, as well as documentation by provider and ancillary staff.

### Data analysis

The dataset was organized into three time periods: March 15th, 2019–December 31st, 2019, March 15th, 2020–December 31st, 2020, and March 15th, 2021–December 31st, 2021 with the latter two containing the time period including the pandemic. Patient demographics and visit characteristics were analyzed using descriptive statistics: (1) median and interquartile range (IQR) for continuous variables and (2) frequencies and percentages for categorical variables. Pearson’s ${\chi }^{2}$ test was utilized for testing associations for sex, ethnicity, GCS, and ISS, while Fisher’s exact test was utilized for age categories, race, and ED disposition. Age (continuous), hospital charges, hospital, and ED LOS were analyzed for differences among the three periods with Kruskal–Wallis test. Percentage changes with 95% confidence intervals were estimated using Poisson regression models with the year as the main predictor, and outcome measures as injury types, injury location, disability or death following injury, incident demographics including intent, location of shooting, relationship of shooter to patient, presence of adult supervision, firearm storage, and the risk factors described above. P values less than 0.05 were considered statistically significant. All analyses were performed using Stata 16.1 (College Station, Texas).

## Data Availability

The datasets generated and analyzed, supporting the conclusions of this article, are not publicly available datasets. The trauma registry is a private institutional database, however, can be made available from the corresponding author on reasonable request and at the discretion of the custodians.

## Abbreviations

ED: Emergency department
POV: Privately owned-vehicles
US: USA
ICD-10: International classification of diseases—tenth edition
ISS: Injury severity score
GCS: Glasgow coma scale
LOS: Length of stay
Obs: Observation
IMU: Intermediate care unit
ICU: Intensive care unit
IR: Interventional radiology
OR: Operating room
AMA: Against medical advice
GSW: Gunshot wound
IQR: Interquartile range
